# Isolated Tuberculosis of the First Metatarsal of the Right Foot Without Pulmonary Involvement: A Rare Case

**DOI:** 10.7759/cureus.42552

**Published:** 2023-07-27

**Authors:** Sankalp Yadav, Gautam Rawal, Madhan Jeyaraman

**Affiliations:** 1 Medicine, Shri Madan Lal Khurana Chest Clinic, New Delhi, IND; 2 Respiratory Medicine & Critical Care, Max Super Speciality Hospital, New Delhi, IND; 3 Orthopaedics, ACS Medical College and Hospital, Dr. MGR Educational and Research Institute, Chennai, IND

**Keywords:** cbnaat/xpert/rif assay, first metatarsal, fifth metatarsal, osteomyelitis treatment, genital tuberculosis, mtb (mycobacterium tuberculosis)

## Abstract

Tuberculosis of the small bones of the foot is a rare clinical condition. Oftentimes, there is a delay in diagnosis, which could adversely affect the results. Isolated cases of tuberculosis of the first metatarsal of the right foot without pulmonary involvement are seldom reported. Herein, a case of a 13-year-old Indian female who presented with complaints of pain, swelling, and discharge from her right foot is presented. A diagnostic workup led to a definite diagnosis of isolated tuberculosis of the first metatarsal of the right foot without pulmonary involvement, and she was put on anti-tubercular treatment.

## Introduction

Tuberculosis is a disease caused by *Mycobacterium tuberculosis* and is a concern for public health [1]. It is usually rampant in countries in Asia, Africa, and Europe [2]. Tuberculosis is one of the most significant contributors to morbidity and mortality [3]. Usually, inhalation of aerosols infected with the bacteria results in infection, but reports of extrapulmonary involvement without any pulmonary seeding are available in the literature [4].

Extrapulmonary tuberculosis constitutes 10-15% of all cases of tuberculosis [5]. Out of these, skeletal involvement is reported in only 1-3% of cases [6]. Osteomyelitis due to *M. tuberculosis* is a rare condition [2]. Furthermore, tubercular involvement of the small bones of the foot is seldom reported [2]. There is a paucity of data related to this infection of small bones [2]. Usually, tuberculosis of the small bones of the foot is seen in the calcaneum, talus, first metatarsal, navicular, and medial and intermediate cuneiforms [7].

Here, the case of a 13-year-old Indian female who came with complaints of pain and discharge from her right foot is presented. An extensive diagnostic workup helped in establishing the diagnosis of isolated tuberculosis of the first metatarsal of the right foot without pulmonary involvement, and she was put on anti-tubercular chemotherapy.

## Case presentation

A 13-year-old Indian female belonging to a low-income family presented with complaints of pain, swelling, and discharge from her right foot for two months. She was asymptomatic until 60 days ago, when she had pain in her right foot's dorsal aspect. This was followed by an insidious onset of swelling over the dorsum of the right foot. Furthermore, for the last month, there have been discharging sinuses from the anteromedial side of the right foot over the head of the first metatarsal and near the attachment of the Achilles tendon over the calcaneum. These sinuses were associated with purulent (non-blood-tinged, yellow-colored, non-foul-smelling) discharge for one month. It was also associated with a limp, as she was unable to bear weight on her right foot.

The pain was sudden in onset and progressive. It was sharp, localized on the right foot (dorsum), aggravated on walking, and relieved partially after consumption of over-the-counter non-steroidal anti-inflammatory drugs. There was no history of constitutional symptoms of tuberculosis or trauma. Moreover, there was no history of tuberculosis in her or in her contacts. She was a migrant from a different state.

A general examination was indicative of a thin-built female with a temperature of 98.4°F, a pulse of 71 beats per minute, a blood pressure of 110/70 mmHg, a respiratory rate of 17 breaths per minute, and an oxygen saturation (SpO_2_) of 99% in room air.

**Figure 1:**
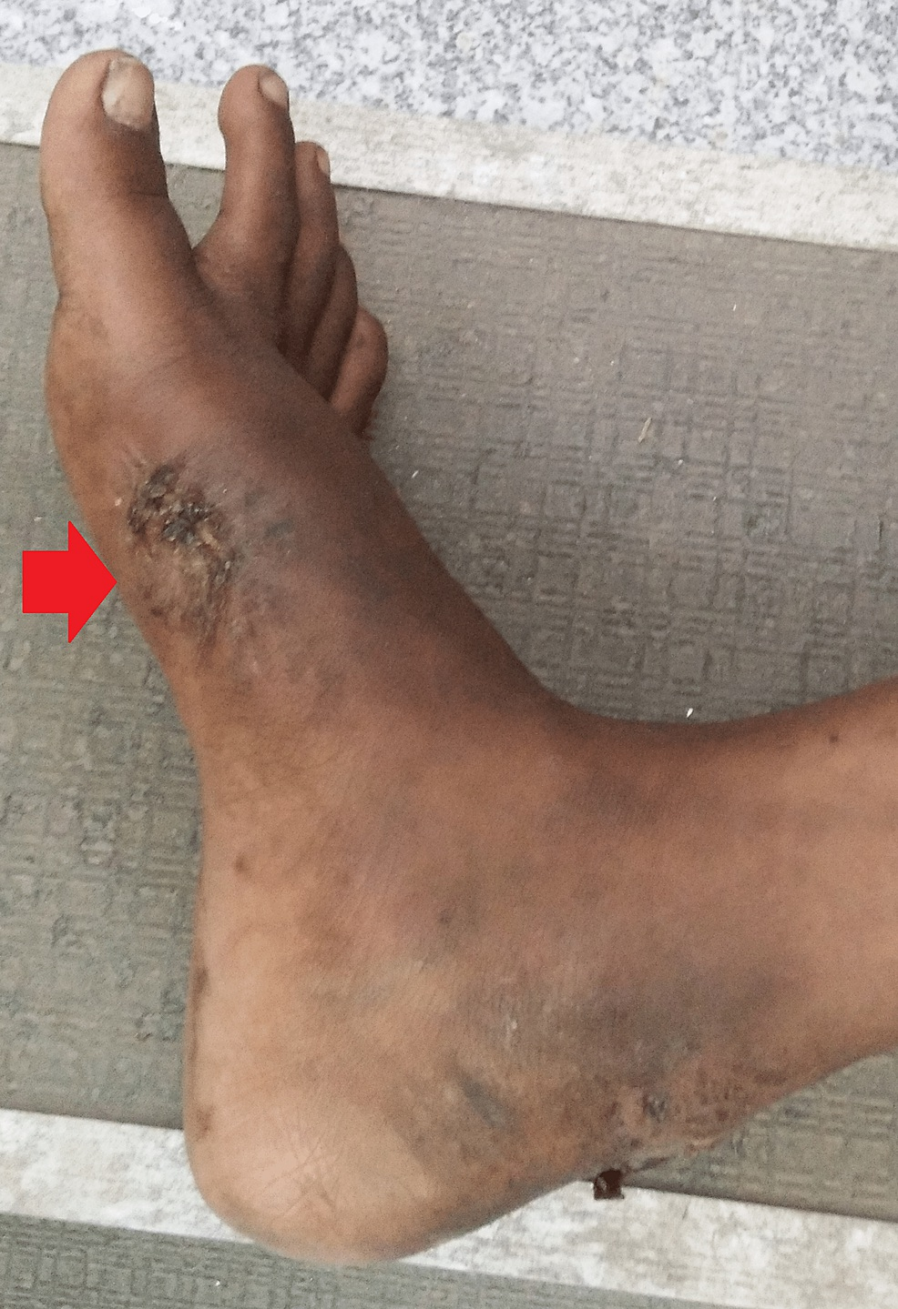
A 5×5 cm wound with an active purulent discharging sinus over the first metatarsal

Local examination of the right foot indicated a 5×5 cm wound with an active purulent discharging sinus over the dorsum lying over the first metatarsal with diffuse edges. This was associated with pain, which led to a decrease in the range of movement of the first metatarsophalangeal joint, eversion and inversion movements of the right foot, and dorsiflexion and plantar flexion (Figure 1).

**Figure 2:**
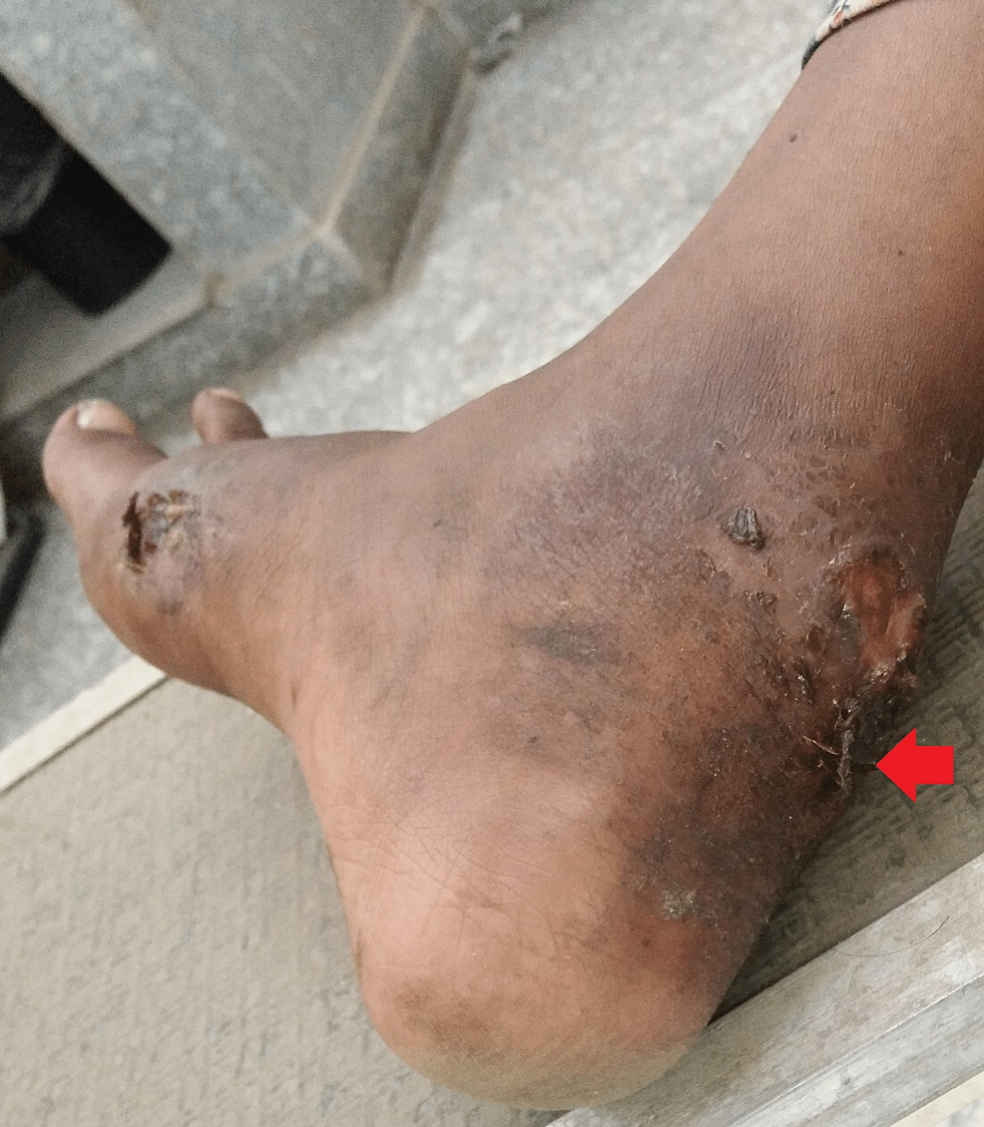
A 5x6 cm wound over the right foot near the attachment Achilles tendon

There was another discharging sinus about 5x6 cm over the right foot near the attachment Achilles tendon over the calcaneum, about 3 cm from the lateral malleolus (Figure 2).

The skin surrounding the sinuses was warm to the touch and erythematous, but there were no dilated veins. Moreover, there was no cyanosis, koilonychia, clubbing, icterus, pallor, or lymphadenopathy. Her systemic examination was unremarkable.

As the initial examination was suggestive of non-specific clinical features, a preliminary diagnosis of pyogenic osteomyelitis was made with differentials for bone tumor, fungal osteomyelitis, tuberculosis, and granulomatous diseases, such as gout and sarcoidosis.

**Figure 3:**
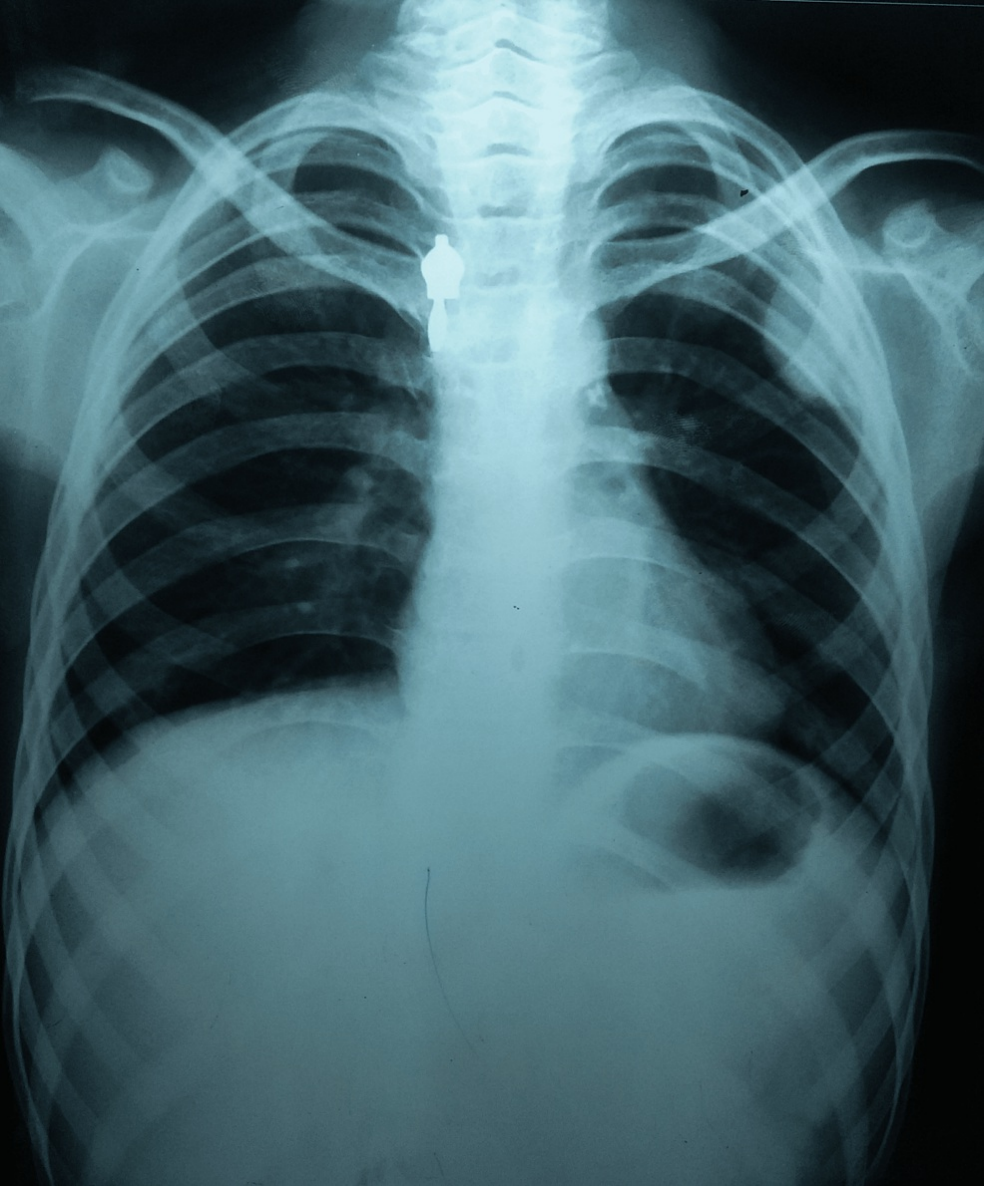
A chest radiograph (P-A) view not suggestive of any pulmonary disease P-A: postero-anterior

A detailed laboratory workup showed that all the serological markers were within normal limits except for an elevated erythrocyte sedimentation rate of 52 mm in the first hour. Her induced sputum for acid-fast bacilli and cartridge-based nucleic acid amplification tests were negative. In addition, her HIV (I and II) was non-reactive, and her hepatitis (A, B, and C) panel was negative. Her chest radiograph was not suggestive of any pulmonary disease (Figure 3).

**Figure 4:**
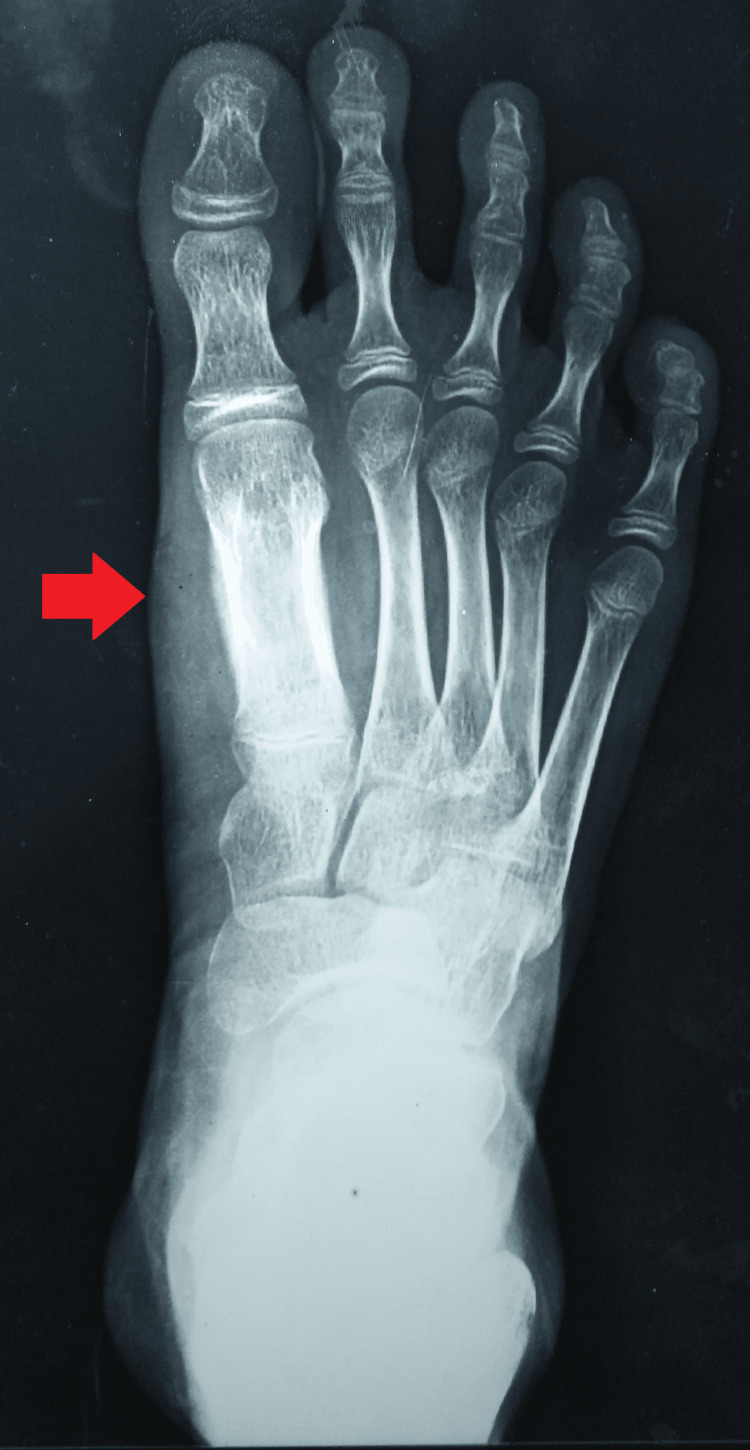
Plain radiograph (A-P) view of the right foot showing soft tissue swelling and involvement of the first metatarsal head A-P: antero-posterior

**Figure 5:**
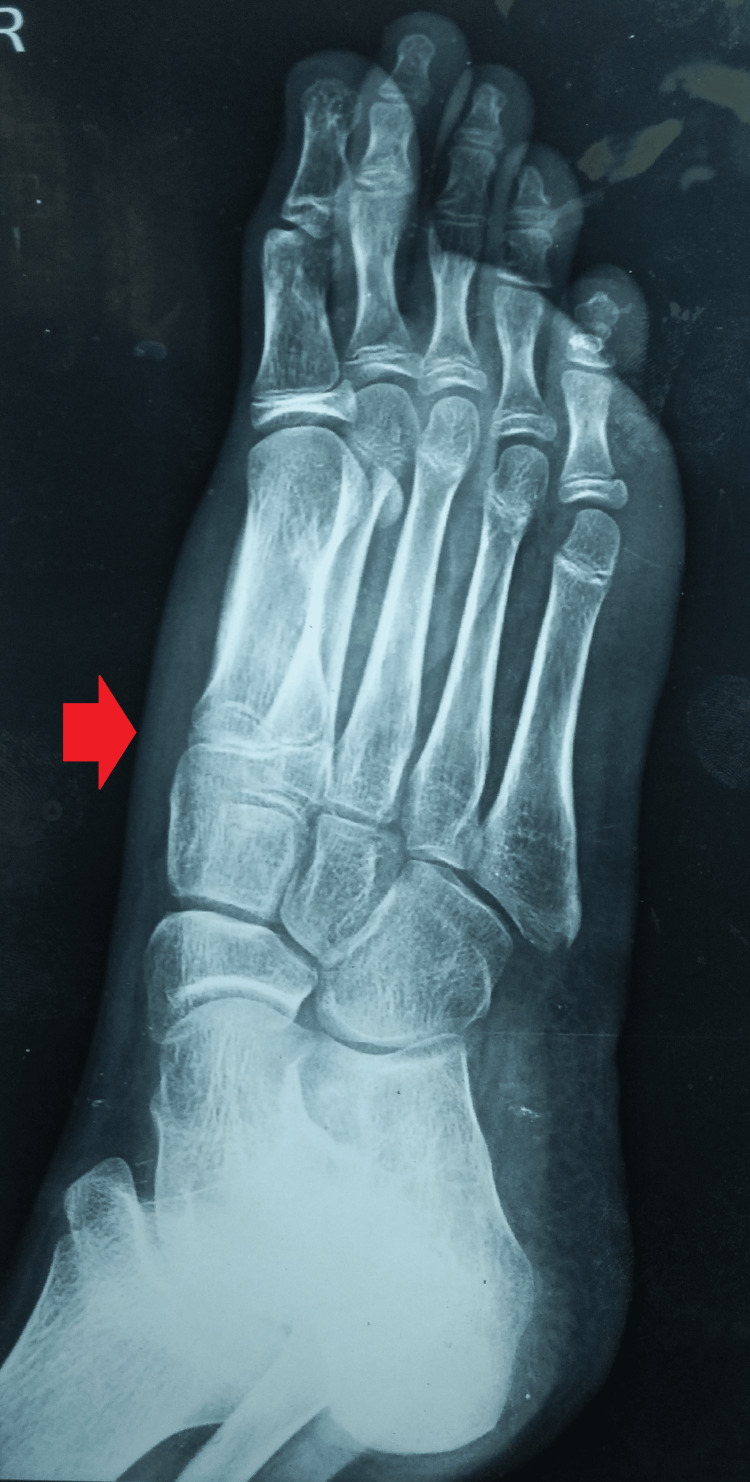
Plain radiograph oblique view of the right foot showing soft tissue swelling and involvement of the first metatarsal head

An anteroposterior and oblique radiograph of the right foot was suggestive of an osteolytic lesion with cortical thinning of the first metatarsal head (Figures 4, 5).

An open biopsy and wound debridement resulted in the drainage of about 10 mL of yellowish-colored pus. Post-curettage, the samples were sent for histopathological and microbiological investigations. The aspirated pus was negative for acid-fast bacilli on Ziehl-Neelsen staining. Histopathology was remarkable for tuberculosis with epitheloid granulomas, caseating necrosis with Langhans giant cells, and lymphocytes. There was a detection of *M. tuberculosis* (low with no resistance to rifampicin) on a cartridge-based nucleic acid amplification test. The same was evident in the on line-probe assay and culture on liquid culture media; however, there was no resistance to any of the first-line drugs for anti-tubercular treatment. Magnetic resonance imaging of the right foot was advised, but the patient was unwilling to undergo the same due to financial constraints, and the diagnosis had already been established by other tests.

The diagnosis of tuberculosis of the first metatarsal of the right foot without pulmonary seeding was made, and she was put on anti-tubercular drugs according to her weight, initially with four drugs for 56 days and followed with three drugs for a period of 10 months (Table 1). A tablet of pyridoxine (1 mg/kg/day) was also added for the full course duration, and dietary advice for a high-protein intake was given.

**Table 1:** Anti-tubercular regimen according to her weight

Phase	Drug	Dose	Duration
Intensive phase	Rifampicin	10 mg/kg	56 days
	Pyrazinamide	25 mg/kg	56 days
	Ethambutol	15 mg/kg	56 days
	Isoniazid	5 mg/kg	56 days
Continuation phase	Rifampicin	10 mg/kg	10 months
	Ethambutol	15 mg/kg	10 months
	Isoniazid	5 mg/kg	10 months

**Figure 6:**
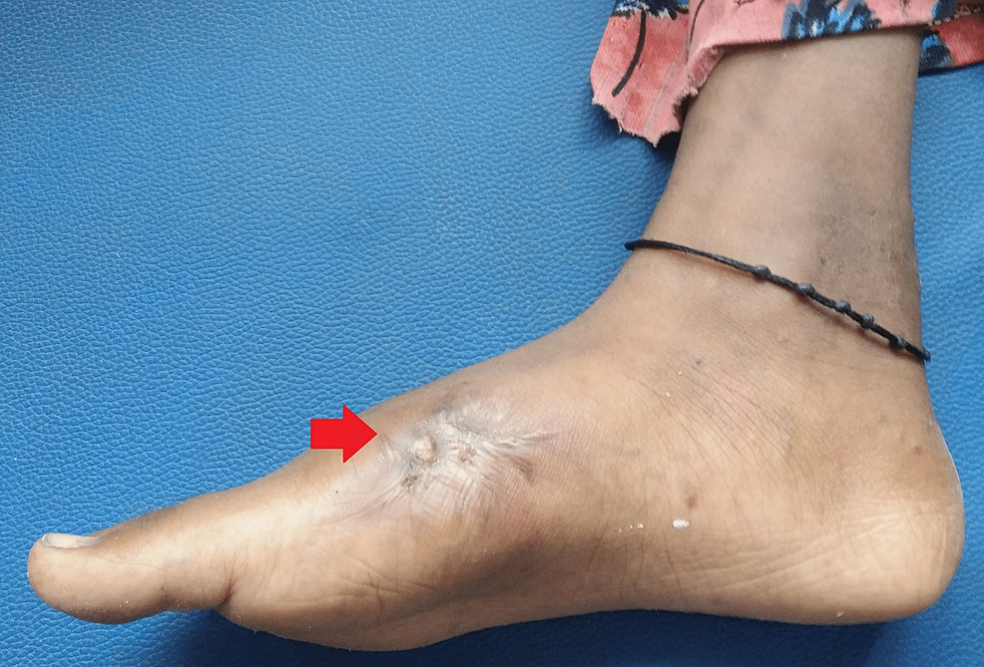
Gross image at the fourth month of follow-up showing a healed discharging sinus

**Figure 7:**
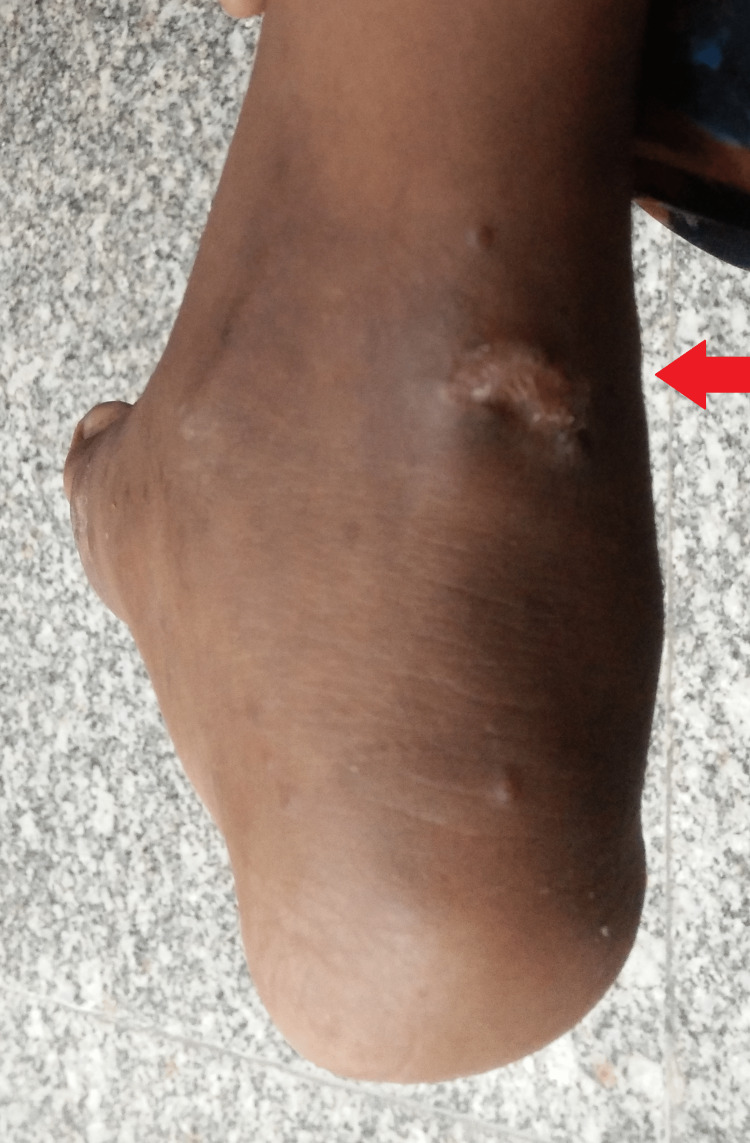
Gross image at the fourth month of follow-up showing a healed discharging sinus

Currently, she has been on treatment for four months with no remarkable adverse drug reactions and is regularly followed up in the infectious diseases and orthopedics outpatient department. There is marked improvement with the healing of both the discharging sinuses and a reduction in swelling and pain (Figures 6, 7).

However, at her parents' request, she was referred to a different district with advice for treatment adherence and regular follow-up in a nearby infectious diseases and orthopedic outpatient department.

## Discussion

Tuberculosis of the bones and joints is rare and is usually reported in less than 3% of total cases of extrapulmonary tuberculosis [2]. Tuberculosis of the small bones of the foot is an extremely rare condition [8]. The incidence of tuberculosis of the metatarsal is less than 0.5%, with the first and fifth metatarsals being most commonly involved [9,10]. Furthermore, isolated involvement without any pulmonary presence of lesions is seldom reported, especially in immunocompetent patients [2].

The diagnosis is extremely challenging [8]. This is mainly due to overlapping clinical features with other musculoskeletal disorders [11]. Patients mostly present with ill-defined symptoms, such as pain, stiffness, and swelling [8]. This condition is paucibacillary, which further delays the diagnosis, as unlike in our case, where the diagnosis was established by cartridge-based nucleic acid amplification test, histopathology, pus culture, and line-probe assay, it requires extensive use of radiometric techniques for a definite diagnosis [8,11]. Lastly, as there is a paucity of literature related to isolated metatarsal tuberculosis, a lack of awareness among the treating physicians also contributes to a delay in management [12]. In addition, the non-availability of free investigations, financial constraints, and non-uniform policies for free slots in radiometric investigations, such as magnetic resonance imaging and computed tomography, result in diagnostic delays.

Isolated tuberculosis of the first metatarsal is limited to a few case reports [2,9,13]. The present case shares similarities with the cases of Madi et al. and Sarwal et al., where patients presented with no lesions in the absence of constitutional symptoms, clinical features like swelling and the presence of a discharging sinus, a raised erythrocyte sedimentation rate, and radiological and histopathological findings [9,13].

Treatment options mainly revolve around conservative management with anti-tubercular drugs [14]. In India, detailed medical management is outlined in the National Tuberculosis Elimination Program (India) guidelines [14]. Surgical maneuvers are used only in advanced cases [14].

Here, an isolated case of tuberculosis of the first metatarsal of the right foot without pulmonary involvement is presented. A detailed diagnostic workup helped in this paucibacillary case even in the presence of constraints in getting radiometric investigations due to financial challenges. This case would serve to motivate the reporting of other similar conditions, mainly from the endemic areas. This will help in modifying or creating new management strategies.

## Conclusions

A case of tuberculosis of the first metatarsal of the right foot without any history of trauma or pulmonary involvement in a 13-year-old female is presented. A battery of laboratory tests helped in the timely initiation of anti-tubercular drugs. The gross images at the fourth-month follow-up clearly justify the prompt management, especially in the background of the non-availability of advanced radiometric investigations due to financial challenges.
